# Investigating SH-SY5Y Neuroblastoma Cell Surfaceome as a Model for Neuronal-Targeted Novel Therapeutic Modalities

**DOI:** 10.3390/ijms232315062

**Published:** 2022-12-01

**Authors:** Pooja Gangras, Valentina Gelfanova, Graham D. Williams, Samuel K. Handelman, Ryan M. Smith, Marjoke F. Debets

**Affiliations:** Lilly Institute for Genetic Medicine, Eli Lilly and Company, Indianapolis, IN 46225, USA

**Keywords:** SH-SY5Y, neuroblastoma, surfaceome, NRCAM, multi-omics

## Abstract

The SH-SY5Y neuroblastoma cells are a widely used in vitro model approximating neurons for testing the target engagement of therapeutics designed for neurodegenerative diseases and pain disorders. However, their potential as a model for receptor-mediated delivery and uptake of novel modalities, such as antibody-drug conjugates, remains understudied. Investigation of the SH-SY5Y cell surfaceome will aid in greater in vitro to in vivo correlation of delivery and uptake, thereby accelerating drug discovery. So far, the majority of studies have focused on total cell proteomics from undifferentiated and differentiated SH-SY5Y cells. While some studies have investigated the expression of specific proteins in neuroblastoma tissue, a global approach for comparison of neuroblastoma cell surfaceome to the brain and dorsal root ganglion (DRG) neurons remains uninvestigated. Furthermore, an isoform-specific evaluation of cell surface proteins expressed on neuroblastoma cells remains unexplored. In this study, we define a bioinformatic workflow for the identification of high-confidence surface proteins expressed on brain and DRG neurons using tissue proteomic and transcriptomic data. We then delineate the SH-SY5Y cell surfaceome by surface proteomics and show that it significantly overlaps with the human brain and DRG neuronal surface proteome. We find that, for 32% of common surface proteins, SH-SY5Y-specific major isoforms are alternatively spliced, maintaining their protein-coding ability, and are predicted to localize to the cell surface. Validation of these isoforms using surface proteomics confirms a SH-SY5Y-specific alternative NRCAM (neuron-glia related cell adhesion molecule) isoform, which is absent in typical brain neurons, but present in neuroblastomas, making it a receptor of interest for neuroblastoma-specific therapeutics.

## 1. Introduction

Current advances in drug discovery center around novel modalities, such as nucleic acid-based therapies, gene therapies, and cell therapies. While these new approaches provide a means for treating diseases in ways that are inaccessible by traditional drugs, they often come with targeting challenges [1]. A lot of the ongoing research in the field of novel modalities is focused on investigating cell type-specific delivery to achieve higher efficacy at lower drug doses while lowering the risk of off-target effects and toxicity. A vast diversity of novel chemistries and ligands are being studied to develop molecules or conjugates that would preferentially accumulate in the tissue of interest [1,2]. Furthermore, different formulations of non-viral delivery systems, such as lipid nanoparticles (LNPs) and exosomes, are being investigated for tissue-specific delivery [3,4]. These non-viral delivery vectors can also be decorated with ligands to cell type-specific receptors, such as small molecules, peptides, or antibodies for effective targeting of specific cell types [5,6]. It is critical to establish relevant in vitro cell culture models for high throughput screening of the uptake and molecular effect of conjugated or packaged novel therapeutics. Furthermore, appropriate cell culture systems can aid in a higher correlation between in vitro and in vivo results, thereby accelerating drug development. To model drug delivery and receptor-mediated uptake in vitro, it is important to determine how the cell surface of the cell line compares to that of the target cell type. 

In this work, we focus on investigating the SH-SY5Y cell line as a model for drug delivery to brain or dorsal root ganglion (DRG) neurons. The SH-SY5Y cell line is a human neuroblastoma cell line derived from the SK-N-SH cell line, which was cultured from the bone marrow biopsy of a 4-year-old female with metastatic neuroblastoma [7]. The SH-SY5Y cells have been widely used to approximate neurons and dissect pathogenic molecular mechanisms underlying neurodegenerative diseases and pain disorders [8,9,10]. While a few studies have conducted total cell proteomics from undifferentiated and differentiated SH-SY5Y cells [11,12], specific profiling of undifferentiated SH-SY5Y cell surfaceomes remains understudied. To our knowledge, only one study has conducted cells-surface proteomics from SH-SY5Y cells, albeit using a different surface protein labeling strategy and proteomics method [13]. While previous studies have conducted proteomics either from brain tissues, DRG neurons, or SH-SY5Y cells, none of the studies have conducted a systematic comparison of SH-SY5Y neuroblastoma cells to either brain or DRG neurons. In the absence of such a comparison, it becomes difficult to identify novel shuttle receptors of interest and to determine if the SH-SY5Y cell line is suitable to study specific receptor-targeted therapeutics. 

To be able to compare the SH-SY5Y cell surfaceome to the cell surface of the brain or DRG neurons, we developed a computational approach to identify the cell surface proteins expressed in the human brain and human DRG neurons using publicly available proteomic and transcriptomic datasets [14,15,16,17]. We then used a surface proteomics approach to delineate the cell surface proteins expressed on SH-SY5Y cells and compared these to brain and DRG neuron membrane proteins (‘common pool’). To investigate the isoforms of the ‘common pool’ surface proteins, we conducted mRNA sequencing followed by isoform quantification. Using the isoform quantification data we identified the ‘common pool’ proteins where the RNA isoform with the highest expression (major isoform) was annotated to be alternatively spliced. To determine if these alternatively spliced major isoforms are translated, we re-mapped the SH-SY5Y surface proteomics data to an isoform-aware version of the Uniprot database. As a result, we validated isoform-specific peptides for an alternatively spliced isoform of NRCAM (Q92823-4), which we further find is detected in SH-SY5Y neuroblastoma cell-surface proteomics but not in the normal brain total proteomics described by Carlyle et al. [14].

## 2. Results

### 2.1. Bioinformatically Inferring Surface Proteins Expressed in Human Brain Neurons and DRG Neurons Using Proteomics and Transcriptomics Data

**Figure 1:**
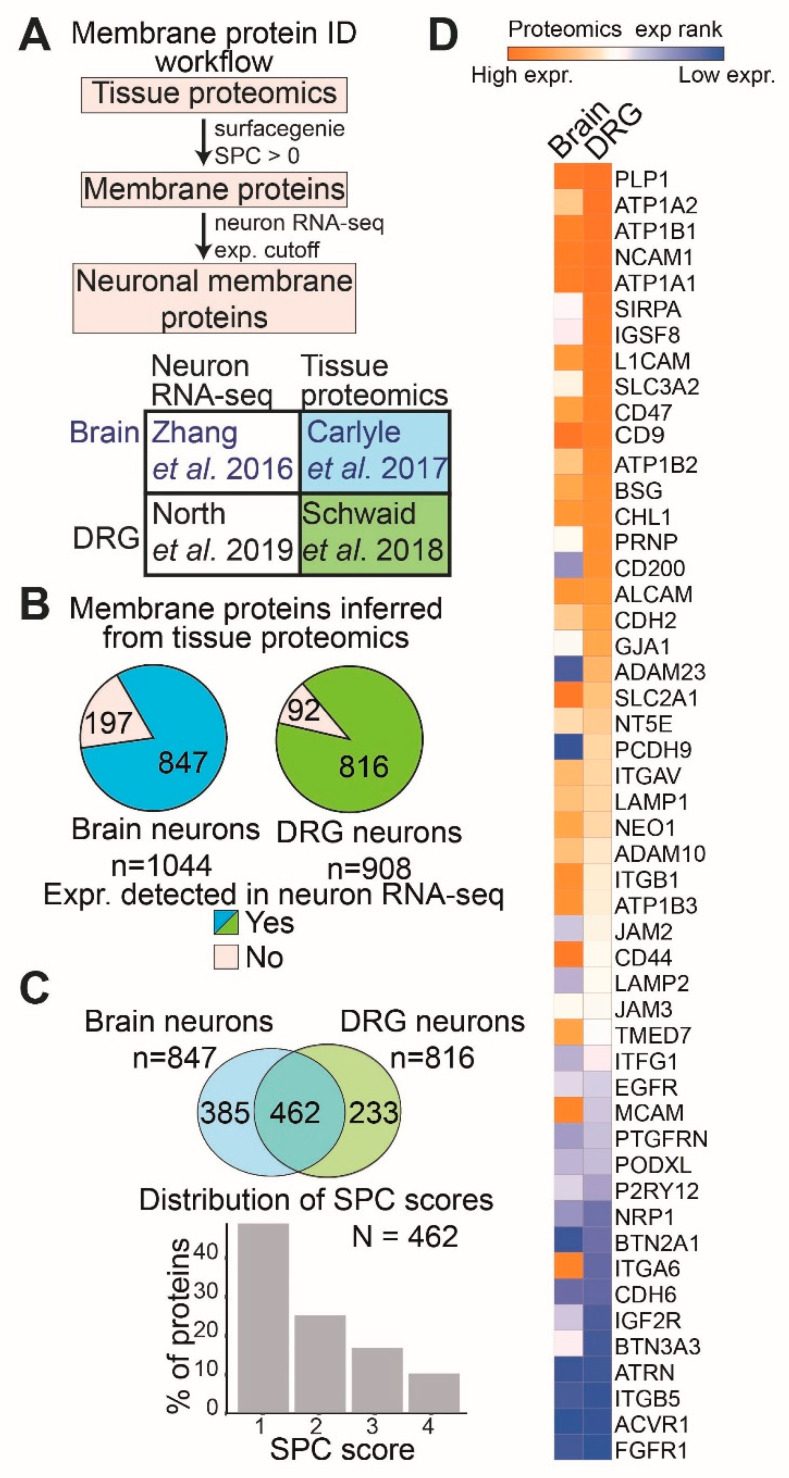
Analysis of human tissue proteomics and neural RNA sequencing data reveals several membrane proteins expressed in both brain and DRG neurons. (**A**) Pipeline for analysis (top) and the datasets used for brain [14,17] and DRG [15,16] neuron inferred membrane proteome analysis (bottom). (**B**) Pie charts representing all the membrane proteins (SurfaceGenie SPC score ≥ 1) identified from the human brain and DRG tissue proteomics. The pie section in green (brain), and blue (DRG) represents a protein with a more than median gene expression (expr.) in brain- or DRG-specific neuronal RNA sequencing. (**C**) Venn diagram comparing the inferred brain and DRG neuronal membrane proteome (top). Bar plot showing the distribution of SurfaceGenie SPC scores for the proteins common to both brain and DRG neuronal membrane proteome. (**D**) Heatmap of the brain and DRG tissue proteomics ranked expression (expr.) of high-confidence (SPC score ≥ 3) surface proteins common to both brain and DRG neuronal membrane proteome. All proteins and their ranks are listed in Table S1.

To determine the expression levels of membrane proteins in brain and DRG neurons, we developed a bioinformatic workflow that combines proteomics, transcriptomic, and membrane protein annotation datasets. We determined the ranked expression of all proteins identified via proteomics from primary tissues and then used the SurfaceGenie [18] Surface Prediction Consensus (SPC) scoring metric to identify membrane-associated proteins at confidence levels ranging from 1–4, where an SPC score of 3–4 indicates a membrane protein most likely expressed on the cell surface (Figure 1A). Next, we filtered the membrane proteins with detectable RNA expression in neuronal cells of the same tissue (Figure 1A). For this workflow, we identified whole tissue proteomics which used either primary human brain regions [14,17] or primary human DRG tissues [15,16], and RNA sequencing datasets which quantified gene expression in neurons isolated by immune-panning/excision from said tissues (Figure 1A). To maintain consistency between the brain proteomic and transcriptomic datasets, we only included the proteomics data generated from the dorsoprefrontal cortex and transcriptomic data generated from cortical neurons [14,17].

We identified 1044 brain membrane proteins and 908 DRG membrane proteins of which 847 and 816 membrane proteins, respectively, were detected in the respective neuronal transcriptomic datasets (Figure 1B). Among these neuronal membrane proteins, 462 are common to both brain and DRG tissues, with nearly 25% of these being high-confidence plasma membrane proteins (SPC score 3–4, N = 50) (Figure 1C, Table S1). Subsequent GO (Gene Ontology) term analysis of these 50 high-confidence surface proteins expressed in both brain and DRG neurons shows that they are involved in many different molecular functions mainly including solute transport and cell adhesion (Figure S1). We then compared the relative proteomics ranked expression of commonly expressed neuronal plasma membrane proteins to find that these membrane proteins show tissue-specific differences (Figure 1D). Where some proteins, such as PLP1, ATP1B1, NCAM1, and ATP1A1, are highly ranked in both DRG and brain tissues, other proteins, such as ITGB5, ACVR1, and FGFR1, show comparatively lower ranks in both. Meanwhile, some proteins, such as CD200, ADAM23, and PCDH9, show a much higher rank in brain neurons than in DRG neurons. These results show that approximately 54% of the membrane proteins expressed in brain neurons are also expressed in DRG neurons, but the expression level of these proteins is regulated in a tissue-specific manner. 

### 2.2. Investigation of the SH-SY5Y Neuroblastoma Cell Surfaceome and Its Comparison to the Inferred Brain and DRG Neuronal Surfaceome

**Figure 2:**
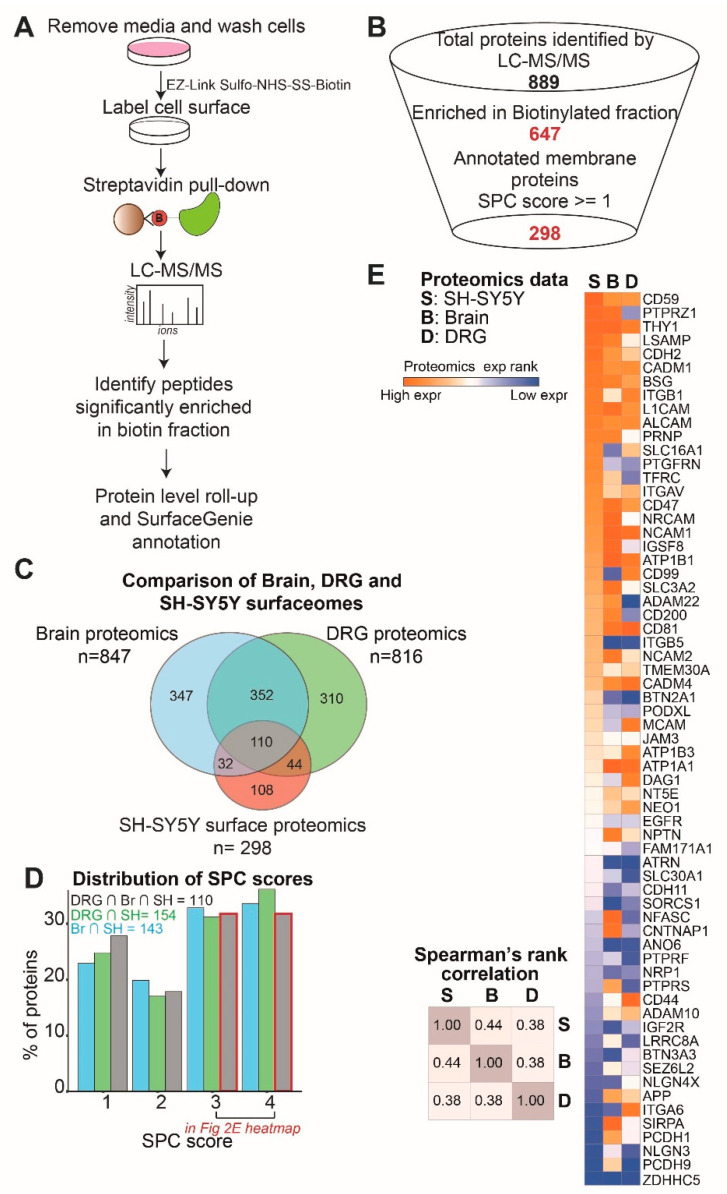
Surface proteomics identifies nearly 300 SH-SY5Y membrane-associated proteins. (**A**) An experimental workflow for cell surface labeling followed by proteomics. (**B**) Bioinformatic selection funnel used to identify biotin-labeled proteins and select for high-confidence surface proteins. Proteins enriched in biotin compared to control fractions listed in Table S2. (**C**) Venn diagram showing the overlap between membrane proteins identified in brain and DRG neurons as well as SH-SY5Y cells. The overlap of each pairwise comparison shown here is statistically significant, as calculated by the hypergeometric test (*p*-values < 2.2 × 10^−16^). All proteins common to all three classes and their expression ranks are listed in Table S3. (**D**) Distribution of SPC scores for pairwise comparisons including SH-SY5Y cells shown in (**C**) and for proteins present in all three classes. (**E**) Heatmap of the brain and DRG proteomics as well as SH-SY5Y surface proteomics ranked expression of high-confidence (SPC score ≥ 3) surface proteins common to all 3 datasets (left). Heatmap of Spearman’s rank correlation for pairwise comparisons of protein expression in the three datasets.

The SH-SY5Y cell line is a human neuroblastoma cell line that is routinely used in neurodegeneration and pain drug discovery research [8,9,10]. To assess the capacity of the SH-SY5Y cell line as a model for receptor-mediated drug delivery and uptake, we investigated SH-SY5Y cell-specific receptors using a surface proteomics approach. We labeled cell surface proteins on live SH-SY5Y cells with biotin, enriched labeled proteins, and then identified these proteins by LC-MS/MS (liquid chromatography with tandem mass spectrometry) for three independently collected biological replicates (Figure 2A). We verified the cell surface labeling approach by assessing the pulldown of a well-known surface protein, TfR1 (transferrin receptor) (Figure S2A).

Peptides identified by LC-MS/MS were mapped to proteins and peptides significantly enriched in the biotin fraction compared to the un-labeled control fraction, and were determined using the limma test [19] (Figure S2B, Table S2). To include only membrane-associated proteins for downstream analysis, we developed a bioinformatic selection workflow that utilizes the SurfaceGenie [18] surface protein annotation scheme. Based on our selection scheme, we define 298 SH-SY5Y membrane-associated proteins (Figure 2B). To further validate the subcellular localization of these proteins, we conducted a GO cellular component term statistical overrepresentation analysis. As expected, we find that the majority of the proteins are linked to terms, such as membrane component, anchoring junction, and plasma membrane (Figure S2C). Thus, using our proteomics approach, we have identified 298 membrane-associated proteins expressed in the neuroblastoma-derived SH-SY5Y cell line. 

While the SH-SY5Y cell line is widely utilized to study molecular mechanisms underlying neuronal disorders, its capability to model receptor-mediated delivery of nucleic acid therapeutics remains largely unknown. We focused on evaluating the potential of SH-SY5Y cells to model the cell surface of brain and DRG neurons, cells primarily implicated in neurodegenerative and pain disorders. To this end, we compared the SH-SY5Y cell surface proteins to the inferred brain and DRG neuronal surface proteins. We find that nearly 60% of the SH-SY5Y cell surfaceome represents surface proteins expressed in both DRG and brain neurons (Figure 2C). Furthermore, nearly 60% of the 110 neuronal proteins commonly expressed in all 3 classes are high-confidence plasma membrane-associated proteins (SPC score 3–4) (Figure 2D, Table S3). We conducted protein class enrichment analysis on the 110 commonly expressed neuronal proteins using PANTHER [20] to find that the enriched classes include cell adhesion molecules, such as integrins and cadherins (Figure S2D). Thus, the majority of the proteins expressed on the cell surface of SH-SY5Y cells are also expressed on the surface of brain and DRG neurons and, among these, cell adhesion molecules are primarily over-represented. 

To determine the cell type-specific differences in the expression of the 110 neuronal proteins commonly expressed in SH-SY5Y cells, brain neurons, and DRG neurons, we compared the ranked protein expression as determined by proteomics (Figure 2E). Upon comparing relative protein expression across the three cell types by Spearman’s rank correlation, we find that the SH-SY5Y cells are slightly more comparable to brain neurons than DRG neurons while being distinct from both (Figure 2E). 

### 2.3. Validation of SH-SY5Y Surface Proteomics by RNA Sequencing and Identification of SH-SY5Y Specific Surface Protein Major Isoforms

Previous studies have shown that genes encoding the spliceosomal machinery are upregulated in high-risk neuroblastoma patient samples when compared to low-risk patient samples, and that high-risk neuroblastoma samples exhibit unique splicing patterns [21,22]. Studies also find that neuroblastoma cells exhibit aberrant and/or alternative splicing due to mutations in intronic splicing motifs [23]. We hypothesized that SH-SY5Y cells, a neuroblastoma cell line, may exhibit changes in splicing patterns compared to brain and DRG neurons, leading to the expression of alternative neuronal surface protein isoforms. Alternatively, long-term cell culture specific changes in alternative splicing may also contribute to changes in isoform expression between SH-SY5Y cells, normal neurons, and other neuroblastoma cell lines. While some previous studies have conducted RNA sequencing using undifferentiated SH-SY5Y cells [24,25], we proceeded to generate mRNA sequencing since we required deep and paired-end sequencing data to reliably quantify isoforms using Kallisto (Table S4). 

We found a high correlation between the gene expression values (TPM—transcripts per million) of two SH-SY5Y RNA sequencing replicates (Figure S3A). Among the 298 SH-SY5Y membrane-associated proteins identified by surface proteomics, RNA expression of 294 was supported by the RNA sequencing data (S3B). Proteins identified by surface proteomics where RNA expression was not detected (four proteins) are either likely encoded by highly unstable transcripts (IGLON5), the peptides mapping to these proteins are similar to other proteins (peptide detected is common to FZD2 and FZD7), or identification of peptides mapped to these proteins is poor (PCDHB10) (Figure S3B). 

Thus, we proceeded to investigate the relationship between RNA and protein expression of the 110 ‘common pool’ surface proteins (Figure 2C). We found that genes expressed over a wide range at the RNA level were also detected by surface proteomics with relatively alike intensity in the biotin fraction compared to the control (Figure S3C). To determine the most highly expressed isoform representing each protein in the ‘common pool’ proteins, we first analyzed the transcriptomic data to identify the major isoform per gene for all genes detected in SH-SY5Y cells by RNA sequencing i.e., isoform with the highest expression as measured in TPM (transcripts per million). Then, we determined the APPRIS (annotating principal splice isoforms) annotations of the major isoforms representing ‘common pool’ proteins and found, as expected, most of the major isoforms to be annotated principal isoforms (Figure S3E). Meanwhile, ~7% of the major isoforms are APPRIS-annotated alternative isoforms, and ~25% are unannotated by APPRIS, thereby indicating that these are un-conserved alternatively spliced isoforms. 

**Figure 3:**
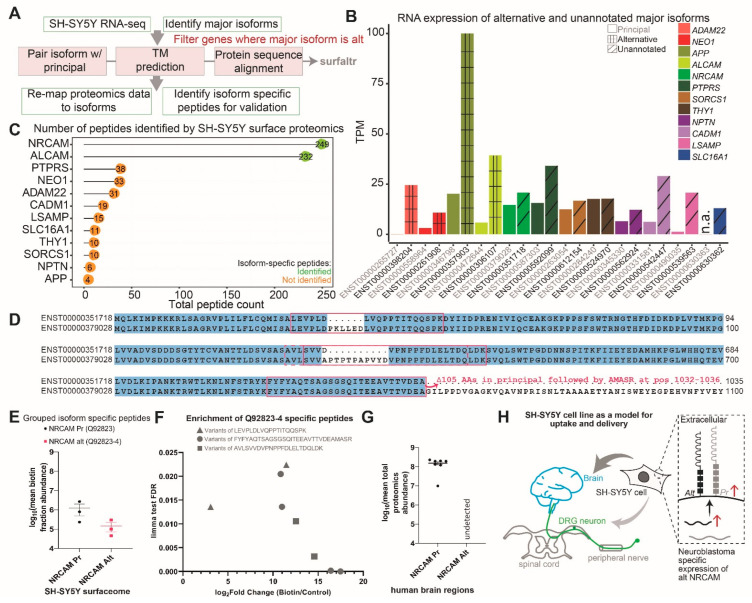
Alternatively spliced major isoforms expressed on the surface of SH-SY5Y cells. (**A**) Schematic describing the bioinformatics pipeline for identification of surface protein isoforms expressed on SH-SY5Y cells by using both proteomics and transcriptomics data. Grey boxes describe all the analyses included in the surfaltr R package (details in the methods section). (**B**) Barplot showing RNA expression of alternatively spliced and APPRIS-unannotated major isoforms for surface protein-coding isoforms expressed in SH-SY5Ycells and common to the brain and DRG tissues. Isoform quantification for all transcripts listed in Table S4. Alternative, principal, and unannotated isoforms are shaded as indicated in the legend. (**C**) Total number of peptides identified via surface proteomics for alternatively spliced and APPRIS-unannotated major isoforms described in (**C**). (**D**) Protein sequence alignment of NRCAM alternatively spliced (NRCAM alt, ENST00000351718) SH-SY5Y specific major isoform to NRCAM APPRIS-annotated principal isoform (NRCAM Pr, ENST00000379028). Regions where the two isoforms differ in sequence have been highlighted by the red box. The red arrow followed by text indicate sequence of the NRCAM alt isoform (ENST00000351718) not shown in the alignment and represented by the peptide indicated by a circle in (**F**). (**E**) Plot showing log10(mean abundance) of NRCAM isoform-specific peptides in the three biotin fraction replicates. Both isoforms are detected at comparable levels in the SH-SY5Y surfaceome dataset. (**F**) Scatterplot showing the enrichment of NRCAM Q92823-4 isoform-specific peptides in SH-SY5Y surface biotinylated fraction compared to control (X-axis) and the statistical significance of this enrichment in three biological replicates, as determined by the limma test FDR (Y-axis). (**G**) Boxplot showing the median abundance of NRCAM isoform-specific peptides in the human brain, as detected by Carlyle et al. [14] using proteomics. Abundance is indicated as mean log10 transformed intensity for all peptides mapping to the NRCAM isoform. Peptides specific to the alternative NRCAM (Q92823-4) were not detected in any brain region (dots represent the eight brain regions tested—dorsolateral prefrontal cortex, primary visual cortex, hippocampus, amygdala, mediodorsal nucleus of the thalamus, striatum, and cerebellar cortex). (**H**) Model summarizing the results of this study, which shows that the SH-SY5Y surfaceome is representative of both brain and DRG neuron surfaceomes, but expression levels are more similar to the brain (indicated by dark and light grey arrows). Furthermore, SH-SY5Y cells express a neuroblastoma-specific alternatively spliced NRCAM isoform (Q92823-4) which shows discordant expression by being the major isoform at the RNA level but the minor isoform at the protein level, as indicated by the red arrows.

We proceeded to further investigate the highly expressed (alternative isoform TPM > 10) major isoforms representing only the high-confidence (SPC ≥ 3) surface proteins among the common pool proteins (N = 12 out of 110, shown in Figure 3B). First, we compared the expression of these alternative isoforms directly to the known principal isoforms of these genes to determine the fraction of gene output represented by each isoform (Figure 3B). In the case of genes, such as *ADAM22*, *NEO1*, *APP*, *ALCAM*, *CADM1*, *LSAMP*, and *SLC16A1*, the alternative isoform expression is significantly higher than the principal isoform. For the rest, that is, *NRCAM*, *PTPRS*, *SORCS1*, *THY1*, and *NPTN*, the expression of the alternative isoform expression is comparable but slightly higher than the principal isoform. To determine the difference in the surface topology of the major alternative isoforms, as compared to the principal isoforms, we analyzed the amino acid sequence of the isoform pairs using surfaltr [26], a program previously published by us for this application (Figure S3E). We find that all the alternative major isoforms are predicted to have transmembrane domains and, hence, predicted to localize to the cell surface (Figure S3E). Alternative isoforms have the same transmembrane topology as the canonical isoform from the same gene, even though most of them (9 out of 13) have a lower number of amino acids, as compared to the principal isoforms (Figure S3E). Thus, if said alternatively spliced isoforms are translated to proteins, then they will contribute to the diversity of the SH-SY5Y cell surfaceome. 

### 2.4. SH-SY5Y Cells Specifically Express an Alternatively Spliced Isoform of NRCAM

We remapped the peptides identified by surface proteomics to an isoform-aware human protein database and found isoform-specific peptides for two proteins, namely NRCAM and ALCAM (Figure 3C). Thus, for NRCAM, the major isoform identified by RNA sequencing (ENST00000351718) is indeed translated to make protein (Q92823-4) that localizes to the cell surface, as predicted by surfaltr. However, for ALCAM, we only detected the peptides specific to the principal ALCAM isoform (ENST00000472644, Q13740) on the cell surface. Hence, even though the ALCAM alternatively spliced isoform appears to be the most highly expressed isoform, its translation and expression on the cell surface are likely highly regulated by other mechanisms that remain unknown. For all other proteins, only peptides common to all isoforms were detected by proteomics and, thus, the specific isoform majorly expressed on the SH-SY5Y cell surface remains undetermined (Figure 3C). 

We find that the SH-SY5Y cell line highly expresses the *NRCAM* transcript. At an isoform level, *NRCAM* alternative transcript (ENST00000351718.8) is the major isoform and is expressed 1.4-fold more than the conserved canonically annotated (APPRIS Principal1) transcript (ENST00000379028.8) (Figure 3B). While the surface topology of NRCAM isoforms is very similar (Figure S3E), aligning their protein sequences using surfaltr revealed three regions where they differ (Figure 3D). In the SH-SY5Y surface proteomics data, both NRCAM principal and alternative isoform-specific peptides are detected (Figure 3E, Table S2). All detected peptides unique to the alternative NRCAM (Q92823-4) are significantly enriched in the biotin fraction compared to the control (Figure 3F). 

Based on proteomics, NRCAM expression in SH-SY5Y cells (proteomics rank 111 of 298) is comparable to brain neurons (proteomics rank 149 of 5039) and not to DRG neurons (proteomics rank 2580 of 5245) (Figure 2E). We hypothesized that the expression of the NRCAM alternative isoform (Q92823-4) is likely neuroblastoma-associated and, thus, specific to the SH-SY5Y cell line. Studies have shown that NRCAM protein is over-expressed not only in low-grade pediatric neuroblastoma patients by immunohistochemistry on tissue microarrays [27], while *NRCAM* mRNA is over-expressed in cell lines derived from human brain tumors as compared to the normal human brain by RT-PCR and northern blots [28]. Furthermore, Sehgal et al. identified an alternative *NRCAM* isoform, shorter than the transcript we found, specific to cell lines derived from human brain tumors and other human cancers [28]. We then investigated the abundance of NRCAM isoform-specific peptides in the human brain tissue proteomics described by Carlyle et al. We found that, as in SH-SY5Y cells, NRCAM principal isoform is highly expressed in all tested human brain regions but, unlike SH-SY5Y cells, the alternative isoform is undetected in the Carlyle et al. dataset (Figure 3G). This could be either because alternative NRCAM (Q92823-4) is not expressed in the human brain or because the peptides unique to this isoform were not well detected in this study.

## 3. Discussion

The SH-SY5Y neuroblastoma cell line has been broadly used as a model for mechanistic studies, and it also presents itself as an attractive model to study receptor-mediated internalization of conjugated or encapsulated novel therapeutic modalities. Despite extensive research being preferred in SH-SY5Y cells, their capacity to model drug delivery to relevant tissues, such as brain and DRG neurons, remains unknown. Here, we have taken a systematic approach to delineate the SH-SY5Y surfaceome and investigate its relationship with the SH-SY5Y transcriptome at isoform resolution. 

### 3.1. Surface Proteins Shared between Brain and DRG Neurons Show Tissue-Specific Differences in Expression Levels

We have developed a method for inferring the relative expression of membrane proteins using tissue proteomics, cell type-specific RNA sequencing, and membrane protein annotation databases (Figure 1A). Upon applying this method to two closely related tissues, namely the brain and DRG, we find 847 and 816 neuronal membrane proteins, respectively (Figure 1B). As expected, we find a statistically significant overlap between brain and DRG neuron membrane proteins, but proteins common to both brain and DRG tissue show differences in relative protein expression in each tissue. While some show consistently higher (e.g., NCAM1) or lower (e.g., ITGB5) relative protein expression, others exhibit tissue-specific differences in relative expression (e.g., CD200 and MCAM) (Figure 1D). The GO term analysis of 50 high-confidence surface proteins shown in Figure 1D shows a significant overrepresentation of proteins with transmembrane transporter activity, cell adhesion function, and ATPase activator functions (Figure S1A). 

### 3.2. Using the SH-SY5Y Neuroblastoma Cell Line as a Model for Receptor-Targeted Drug Delivery Needs to Be Evaluated on a Receptor-by-Receptor Basis

We conducted surface biotinylation followed by mass spectrometry to identify SH-SY5Y plasma membrane-associated proteins. To our knowledge, only one study has unbiasedly profiled SH-SY5Y cell surfaceome, albeit using an irreversible labeling reagent, sulfo-NHS-LC-biotin [13]. Meanwhile, our study uses a reversible labeling reagent, sulfo-NHS-SS-biotin, which aids in the specific elution of labeled proteins while reducing the elution of non-specific proteins. Using our approach, 46% of proteins enriched in the biotin fraction are annotated as membrane proteins, while the approach used by Garcia et al. led to 18% enrichment of plasma membrane-associated proteins in the biotin-enriched fraction. To study high-confidence surface proteins, we developed a rational scheme of selection using four surface protein annotation and/or prediction databases summarized by SurfaceGenie [18]. We compared all SH-SY5Y membrane proteins to the inferred brain and DRG membrane proteome and found 65 high-confidence surface proteins common to all 3 cell types. Comparison of proteomics expression ranks between the three tissue classes shows that the SH-SY5Y cell surfaceome is more comparable to that of brain neurons than DRG neurons while being distinct from both. The SH-SY5Y cells likely exhibit these changes in surface protein expression levels as compared to normal neurons, either due to cell-culture-induced artifacts or due to neuroblastoma-driven changes in gene expression. Thus, even if a brain or DRG neuron receptor of interest is expressed in the SH-SY5Y cell line, the differences in expression levels are an important factor to consider when testing the efficiency of receptor-mediated delivery of therapeutics.

### 3.3. Investigation of Alternatively Spliced Surface Protein Isoforms in SH-SY5Y Cells Reveals NRCAM as a Putative Delivery Target for Neuroblastoma

Neuroblastoma develops from the cancerous transformation of nerve cells in the body and, thus, we expect these cells to express a different repertoire of transcripts than those expressed by brain or extra-cranial (dorsal root ganglion) neurons. In this study, we used multi-omic approaches to identify the cell surface protein isoforms which likely give the SH-SY5Y cells a unique identity when compared to the brain and DRG neurons. In the case of surface proteins detected in SH-SY5Y cells as well as brain and DRG neurons, we first identified the most highly expressed isoform at an mRNA level (we refer to this transcript as the major isoform). Interestingly, nearly 32% of these major isoforms are alternatively spliced, of which 7% are APPRIS-annotated and, hence, cross-species conserved, while 25% are APPRIS-unannotated, likely due to lack of conservation. We used transmembrane topology prediction to show that the protein-coding alternatively spliced major isoforms are localized to the cell surface, as in the case of the canonical isoform of the gene. Furthermore, we re-mapped the surface proteomics data to an isoform-aware human proteome to identify the surface-expressed isoforms of NRCAM and ALCAM. Our study validates the expression of NRCAM isoform 4 (Q92823-4) on the SH-SY5Y cell surface, an isoform that remains unexplored concerning neuroblastoma. The NRCAM over-expression has been associated with cancer progression and metastasis [29,30], but which isoform of NRCAM is responsible for this observed effect remains understudied. An alternatively-spliced NRCAM isoform, shorter than the transcript we identified, was found to be specifically expressed in cell lines derived from human brain tumors and other human cancers [28]. Whether this tumor cell line-associated short *NRCAM* isoform is expressed as a protein remains unknown. 

Taken together, these results show that neuroblastoma cells express alternatively spliced NRCAM which is likely not expressed in the normal human brain. The alternative isoform presents extracellular regions unique from the principal isoform. This likely defines a unique identity for neuroblastoma cells when compared to brain neurons despite the overall similarity between the surface proteome of the two cell types. Hence, alternatively spliced NRCAM isoforms may present a novel opportunity for targeting neuroblastoma cells using, for example, receptor-targeted nanoparticles containing cancer suppressor miR-186, which has been shown to inhibit tumor growth and immune escape in neuroblastoma [31]. Further validation of the NRCAM alternative isoform protein in neuroblastoma patient samples as well as research on NRCAM-specific ligands and antibodies that trigger internalization will further strengthen the case for alternative NRCAM as a delivery target for neuroblastoma. 

Our study presents a novel methodology that combines surface proteomics, bioinformatics, and transcriptomics for the identification of cell type-specific surface protein isoforms. This method can be further expanded and applied to other model systems to delineate ideal delivery anchors on the cell surface. A systematic screen of cell type-specific alternative surface protein isoforms will be critical for designing targeting ligands, such as antibodies, aptamers, peptides, small molecule ligands, etc. which can be used to achieve highly specific delivery of next-generation gene and cell-based therapeutics. Additionally, our methodology could be used to differentiate between secreted and membrane-bound isoforms encoded by a gene to inform the design of RNA therapeutics that target only one isoform.

## 4. Materials and Methods

### 4.1. Cell Culture

The SH-SY5Y cells (#CRL-2266) (ATCC, Manassas, VA, USA)were derived from the SK-N-SH neuroblastoma cell line [32]. The SH-SY5Y cells were cultured in 75 cm^2^ tissue culture plates using standard media conditions (HAMs 1:1 F12 mixture (Gibco, Waltham, MA, USA), 1% MEM NEAA (Gibco, Waltham, MA, USA), 10% FBS (Gibco, Waltham, MA, USA), 1% P/S antibiotics (Gibco, Waltham, MA, USA)).

### 4.2. Cell Surface Protein Labeling and Immunoprecipitation

The SH-SY5Y cells were cultured in 6-well plates using standard media conditions. Upon reaching 90% confluency, the media was aspirated, and cells were washed twice with ice-cold phosphate-buffered saline (PBS). The surfaces of the cells were labeled by incubation with 0.4 mM EZ-Link™ Sulfo-NHS-SS-Biotin (ThermoFisher Scientific, Waltham, MA, USA) in PBS FBS (Gibco, Waltham, MA, USA), and control wells were incubated with PBS alone at 4 °C for 30 min with gentle shaking. Residual biotin was quenched by washing three times with 100 mM glycine in PBS on ice. Control and biotinylated cells were collected by centrifugation at 300× *g* for 5 min. Cell pellets were immediately frozen at −80 °C. 

For immunoprecipitation, cell pellets were resuspended with 0.5 mL of ice-cold RIPA buffer per million cells (Pierce, ThermoFisher Scientific, Waltham, MA, USA) containing protease inhibitor (Sigma-Aldrich, Burlington, MA, USA), then vortexed and incubated on ice for 30 min. When needed, the lysate was pipetted several times to dissolve the pellet. The cell lysates of each fraction were cleared by centrifugation at 10,000× *g* for 10 min at 4 °C, and the supernatants were collected into new low protein binding 2 mL tubes. A BCA assay (ThermoFisher Scientific) (using freshly made standards) was carried out to determine the lysate concentrations to maintain equal protein input across biological replicates. Then, 75 µL of streptavidin magnetic beads (washed as per manufacturer’s instructions with TBST (1X TBS + 0.1% tween-20)) (ThermoFisher Scientific, Waltham, MA, USA) were added to both biotin and control fractions. The tubes were then at room temperature for 1 h. The beads were collected using a magnetic rack, and the supernatant was saved in separate tubes. Magnetic beads were washed three times with 300 µL of RIPA buffer followed by three washes with 300 µL of 0.5 M NaCl in RIPA buffer, and then three washes with PBS. Elution was achieved by reduction in 100 µL of 50 mM DTT (ThermoFisher Scientific, Waltham, MA, USA) in water at RT for 1 h with end-to-end nutation. Beads were collected on a magnetic rack, and eluate was collected in fresh 2 mL tubes. This protocol was conducted for three independently collected biological replicates.

### 4.3. Sample Preparation for Mass Spectrometry Analysis

Eluates were transferred to 0.2 mL 96-well plates and dried under nitrogen. Subsequently, 100 µL of 50% acetonitrile, 1% iodoethanol, and 0.25% triethylphosphine in 50 mM ammonium carbonate were added to the samples for 1 h at 37 °C. After nitrogen-drying, 100 µL of (protein amount/50) trypsin in 100 mM ammonium bicarbonate was added at 37 °C overnight. Detergent removal from digested samples was performed with five cycles of ethyl acetate extraction procedure [33]. The aqueous phase of samples was dried under nitrogen, reconstituted in 100 µL of 0.1% formic acid, and then desalted using ZipTips (Millipore, Burlington, MA, USA). ZipTips (Millipore, Burlington, MA, USA) were prewetted with three aspirate/dispense cycles of 50% acetonitrile and 0.1% TFA and equilibrated with three rinses of 1% acetonitrile and 0.1% TFA. Peptides were captured by 20 aspirate/dispense cycles, washed, and eluted with 50% acetonitrile and 0.1% TFA. Eluates were nitrogen-dried and reconstituted in 20 µL of 1% acetonitrile and 0.1% TFA.

### 4.4. Mass Spectrometry Peptide Analysis

Samples were analyzed with an Orbitrap Fusion™ Lumos™ mass spectrometer using a Thermo-Easy liquid chromatograph high-performance liquid chromatography system and a PepMap™ 3 mm, 100 A, and a 75 µm × 15 cm C18 column (ThermoFisher Scientific, Waltham, MA, USA). The emitter tip temperature was set at 30 °C. The electrospray potential was +1.9 kV and the capillary temperature was 275 °C. Solvent A was 0.1% formic acid (A), and solvent B was 80% acetonitrile and 0.1% formic acid. The gradient was for 120 min using a flow rate of 250 nL/min, starting with 5% B, 32 min 5–45% B ramp, 1 min 45–95% B ramp, and 2 min hold at 95% B for the 35 min method, or starting with 5% B, 87 min 5–45% B ramp, 1 min 45–95% B ramp, and 2 min hold at 95% B for the 90 min method. The instrument was run from 350–2000 *m/z* at 120,000 resolution (maximum injection time of 50 ms). Data-dependent fragmentation was performed with cycle time mode with 3 s between master scans at a HCD collision energy of 25% and orbitrap scan resolution of 30,000. The peptides were identified by searching spectra against both the isoform-agnostic and isoform-aware Uniprot proteomics database [34]. The fixed modification was iodoethanoylation of cysteines. Variable modifications were the oxidation of methionines, deamidation of asparagines/glutamines, and biotinylation (+145.0197) of lysines. Peptide identification and intensity calculation were conducted using an internally validated and routinely used pipeline that has been previously published [34]. Briefly, identification filters were 10 ppm for the parent, a false discovery rate of 10%, and at least two peptides/protein matching. Peptides were integrated with a 1 *m/z* radius and a 2 min window. Ion intensity was estimated by calculating the area under the curve (AUC), as described in Higgs et al. [34]. 

### 4.5. Mass Spectrometry Bioinformatic Analysis

Peptides identified in the control and biotin fraction for all replicates were analyzed using the limma test [19,35], where the peptides enriched in the biotin fraction compared to control were determined by the limma test FDR < 0.05 and log biotin/control fold change ≥ 2. Proteins were defined as being significantly enriched in the biotin fraction compared to the control fraction if at least 30% of the detected peptides of the protein were significantly enriched per the cutoffs described above. Among the proteins enriched in the biotin fraction, high-confidence membrane proteins were identified using SurfaceGenie annotation of SPC ≥ 1 [18]. While a lenient cutoff was utilized for peptide identification, after this complex selection and filtering approach we determined that enriched peptides mapping to high-confidence membrane proteins (N = 298 in Figure 2B) had a peptide identification FDR of 1%, thereby providing confidence in our enrichment and filtering approaches. We provide the 10% FDR peptide level data (Table S2) as supplementary information to allow for exploratory research from this dataset.

The SH-SY5Y protein expression was ranked based on the sum of biotin fraction log2 (AUC) of all significantly enriched peptides per protein.

### 4.6. Western Blot Analysis

Cell pellets were resuspended in RIPA buffer (Pierce, ThermoFisher Scientific, Waltham, MA, USA) and 1X LDS buffer (Pierce ThermoFisher Scientific). Samples were heated at 95 °C for 2 min before being loaded onto a 4–20% PROTEAN^®^ TGX Precast protein gel (BioRad Laboratories, Hercules, CA, USA). Proteins were transferred to a nitrocellulose membrane using the Trans-Blot Turbo Transfer System (BioRad Laboratories). The membrane was blocked using Intercept^®^ blocking buffer (LI-COR, Lincoln, NE, USA). The membrane was incubated with primary antibodies (diluted in Intercept^®^ T-20 antibody diluent, LI-COR) at 4 °C overnight followed by three washes with 1X TBST (ThermoFisher Scientific, Waltham, MA, USA), followed by incubation at room temperature with secondary antibody (diluted in Intercept^®^ T-20 Antibody diluent, LI-COR) for 1 h. Post incubation, the blot was washed three times with TBST and imaged using the Chemidoc XRS system (BioRad Laboratories, Hercules, CA, USA).

#### 4.6.1. Primary Antibodies Used

Transferrin receptor monoclonal antibody (H68.4) (#13-6800, ThermoFisher Scientific). β-actin antibody (#34967, Cell Signaling Technology, Danvers, MA, USA) was used in this study. 

#### 4.6.2. Secondary Antibodies Used

In this study, IRDye 800CW Goat anti-Rabbit IgG (Licor) and IRDye 800CW Goat anti-Mouse IgG (Licor) were used as secondary antibodies.

### 4.7. SH-SY5Y RNA Sequencing Sample Preparation and Data Analysis

The SH-SY5Y cells were cultured in 75 cm^2^ tissue culture plates using standard media conditions. Upon reaching 90% confluency, media was aspirated, and cells were washed once with ice-cold PBS and then harvested with TrypLE (Gibco, Waltham, MA, USA). Following collection, cells were pelleted by centrifugation 1500× *g*, for 5 min, at 4 °C. After pelleting, cells were washed 1× in ice-cold PBS and again pelleted by centrifugation 1500× *g*, for 5 min, at 4 °C. Following pelleting, RNA was isolated using TRIzol reagent (Ambion, Austin, TX, USA). Here, 1 mL of TRIzol was added to cell pellets, triturated, and RNA extracted according to the manufacturer’s suggested protocol including isopropanol precipitation and 70% EtOH wash. Following resuspension of the RNA, RIN was assessed by Agilent 2100 Bioanalyzer with the Agilent RNA 6000 Nano before submission for sequencing at the Beijing Genomics Institute (BGI). Two independently thawed, passaged, and harvested cell populations were used for transcriptomic analysis. Here, RNA sequencing was conducted for two biological replicates.

### 4.8. SH-SY5Y RNA Sequencing Library Preparation, Sequencing, and Data Analysis

The TruSeq Stranded mRNA library (Illumina, San Diego, CA, USA) construction method was used for library preparation. For the Truseq Stranded mRNA protocol, total RNA was used as input for Illumina’s Truseq Stranded mRNA library preparation kit. Here, oligo(dT)-attached magnetic beads were used to purify mRNA. Purified mRNA was fragmented into small pieces with fragment buffer at the appropriate temperature. Then, first-strand cDNA was generated in first strand reaction system by random hexamer-primed reverse transcription, and the second-strand cDNA was generated as well. The reaction product was purified by magnetic beads, afterwards, and A-tailing mix and RNA index adapters were added by incubating to end repair. The cDNA fragments with adapters obtained from the previous step were amplified by several rounds of PCR, and the product was purified by Ampure XP Beads (Beckman Coulter, Pasadena, USA). The libraries were assessed for quality and quantity via the following two methods: (1) by checking the distribution of the fragment size using the Agilent 2100 Bioanalyzer, and (2) by quantifying the library using real-time quantitative PCR (qPCR) (TaqMan Probe, ThermoFisher Scientific). The qualified libraries were amplified on cBot System (Illumina) to generate the cluster on the flow cell. Libraries were sequenced on an Illumina NovaSeq 6000 platform paired-end with paired-end 150 bp sequencing to obtain approximately 100 million reads per library/sample. Data passed the BGI internal data QC before being delivered. The RNA sequencing reads were transferred back to Eli Lilly and Company, and read QC was performed. Short read data was mapped to GRCh38 from Ensembl and summarized at both the transcript level and rolled up to the gene level. Kallisto [36] was used to assemble and quantify the expression of Ensembl-annotated isoforms/transcripts, using default parameters.

### 4.9. Major Isoform Calculation and Membrane Topology Benchmarking

Kallisto isoform level TPM (transcripts per million) values were used to determine the major isoform per gene i.e., the isoform with the highest TPM value. All major isoforms were annotated using APPRIS, and isoforms annotated as alternative or unannotated by APPRIS were prioritized for downstream studies. The list of alternative/unannotated major isoforms of SH-SY5Y cells that encode surface proteins (SPC > 0) were benchmarked against their known principal isoforms using surfaltr [26]. 

### 4.10. Brain and DRG Transcriptomic and Proteomic Datasets

The published datasets used in this study are as follows: Zhang et al. for human brain neuronal RNA sequencing [17], Carlyle et al. for brain tissue proteomics [14], North et al. for DRG neuronal RNA sequencing [15], and Schwaid et al. for DRG proteomics [16] (Figure 3A). Highly expressed genes detected in neuronal RNA sequencing datasets were determined by applying an expression cutoff of more than the median expression in the dataset.

### 4.11. Brain and DRG (Dorsal Root Ganglion) Tissue Datasets Analysis

A ranked protein expression table was obtained for DRG tissue and generated for SH-SY5Y as well as brain proteomics datasets. The proteomics data was filtered to only include membrane proteins (SurfaceGenie [18] Surface Prediction Consensus (SPC) score > 0). Next, neuron-specific proteins were prioritized by selecting proteins with more than the median gene expression in the neuronal RNA sequencing dataset. 

### 4.12. Surface Protein Selection 

Given a set of genes, an in-house R-script was used to identify UniProt IDs followed by matching to the SurfaceGenie annotation [18]. 

### 4.13. GO Term Enrichment Analysis

In Figure 2D, proteins were classified by panther GO biological component enrichment analysis, and significantly enriched protein classes were identified by using Fisher’s exact test followed by Bonferroni multiple testing for FDR calculation. The GO terms were further consolidated using the rrvgo R package [37] to create a treemap plot where the sizes of the terms are proportional to −log10(FDR). In Figure 3C and Figure S3C,D, proteins were classified by PANTHER [20] protein class enrichment analysis, and significantly enriched protein classes were identified by using Fisher’s exact test Bonferroni multiple testing for FDR calculation. 

## Data Availability

The brain and DRG tissue transcriptomic and proteomic data presented in this study are openly available in Carlyle et al. [14] and Schwaid et al. [16] The SH-SY5Y surface proteomics and RNA sequencing processed data presented in this study are available in Tables S2 and S4, respectively.

## Supplementary Materials

The following supporting information can be downloaded at: https://www.mdpi.com/article/10.3390/ijms232315062/s1.
