# Supplementary material for: Investigating SH-SY5Y Neuroblastoma Cell Surfaceome as a Model for Neuronal-Targeted Novel Therapeutic Modalities
Source: Int J Mol Sci. 2022 Dec 1;23(23):15062. doi: 10.3390/ijms232315062 (PMC9739866; doi:10.3390/ijms232315062)
Supplement: Supplementary file 1 [file ijms-23-15062-s001.zip › Supplementary-Figures.pdf]

A

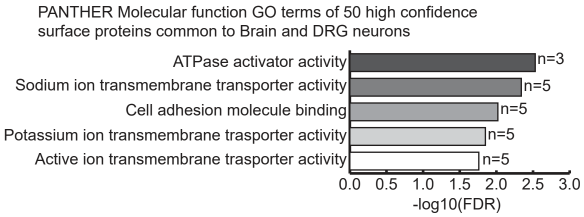

Figure S1 (for Figure 1)

A Barplot showing PANTHER Molecular function GO term enrichment for high confidence surface proteins common to DRG and Brain

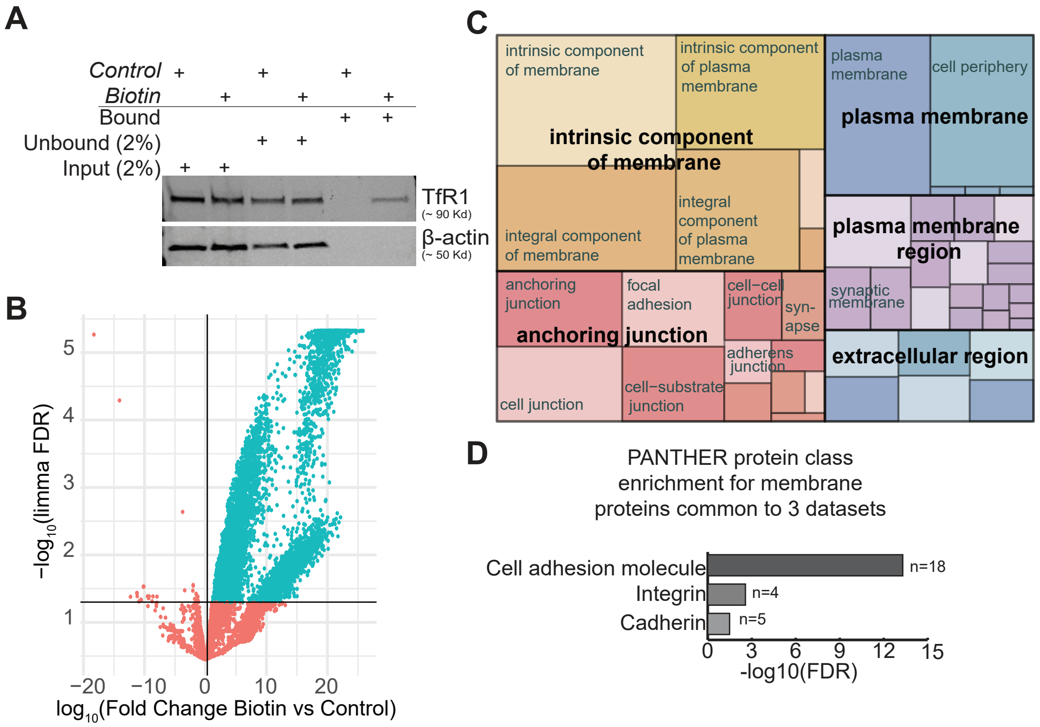

Figure S2 (For Figure 2) Membrane proteins expressed in Brain neurons, DRG neurons and SH-SY5Y cells show a significant overlap primarily represented by adhesion molecules.

A Western blot validating streptavidin pulldown of a well-known surface protein, TfR1, from SH-SY5Y cells post biotin labelling. Lanes are labelled on image.

B Volcano plot showing the $\log_2$ fold change in $\log_2$AUC values (X-axis) for all detected ions in biotin fraction compared to control as well as their -log10(limma test FDR) (Y-axis) when compared across three replicates.

C Tree-map plot summarizing the GO terms statistically overrepresented in the ~300 SH-SY5Y membrane proteins.

D Barplot showing PANTHER protein class enrichment for membrane proteins common to all 3 datasets.

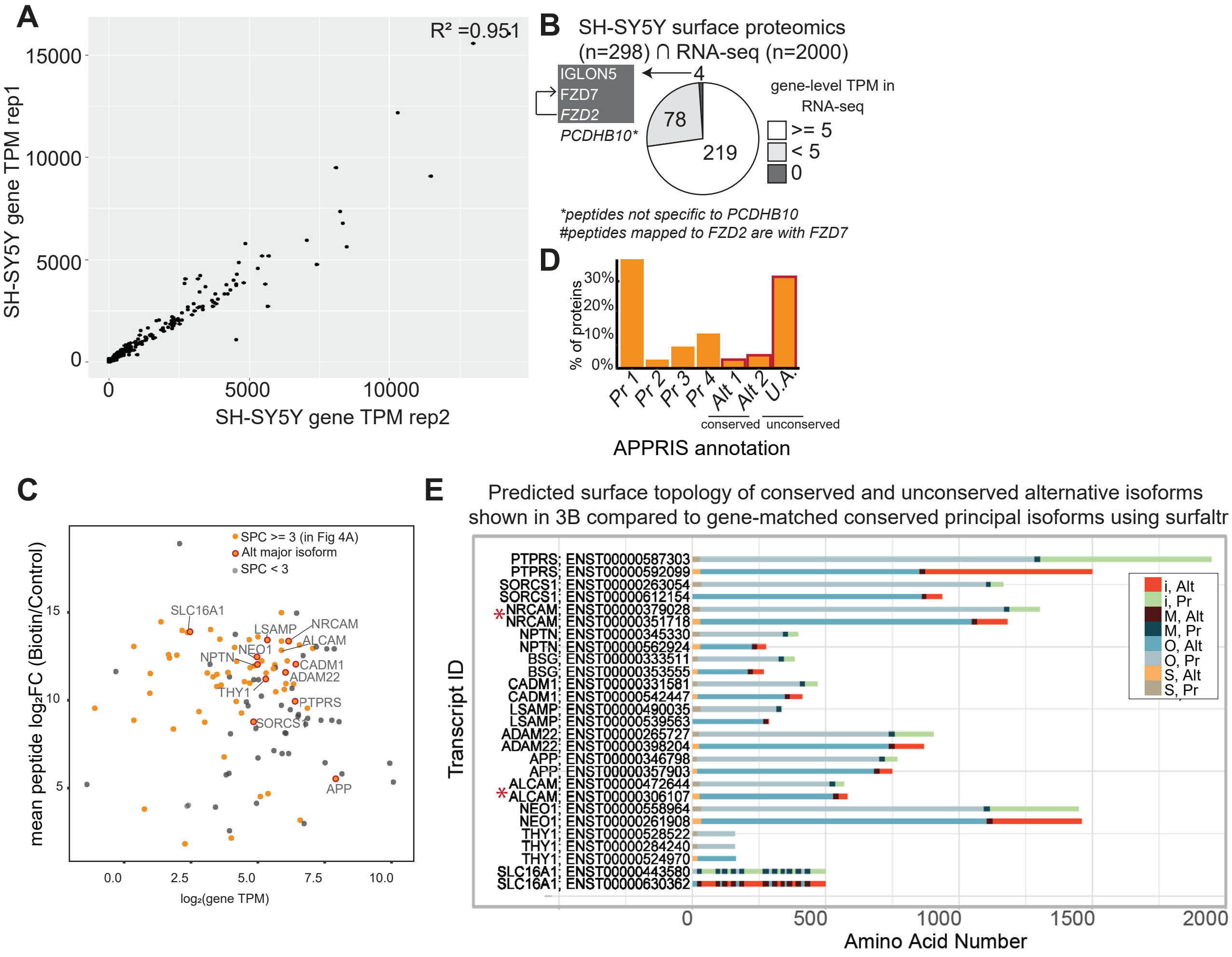

Figure S3 (for Figure 3)

A Scatter plot showing the gene-level TPM values for all genes in both SH-SY5Y RNA-seq replicates.

B Pie chart classifying the gene-level RNA expression of 298 membrane proteins identified by surface proteomics.

C Scatter plot showing the gene-level TPM distribution for the proteins identified by SH-SY5Y surface proteomics which are also expressed in brain and DRG neuronal membrane proteome. (SPC >=3 shown in orange and SPC < 3 shown in grey). Genes highlighted with a red outline encode SH-SY5Y major isoforms that are annotated to be alternatively spliced in Fig S3D.

D Barplot showing the distribution of APPRIS annotation for major isoforms encoding SH-SY5Y membrane proteins which are also expressed in brain and DRG neuronal surface proteome.

E Horizontal barplot showing the transmembrane topologies of isoform pairs shown in Fig 3B as predicted by surfaltr. Red asterix mark proteins where isoform specific peptides were validated by surface proteomics as shown in Fig 3C.
